# Immune-programmed cell death crosstalk implicates plasma sTNFR1 as a candidate biomarker for sepsis-associated encephalopathy

**DOI:** 10.3389/fneur.2026.1954784

**Published:** 2026-10-14

**Authors:** Che Wang, Tong Xin, Yifei Chen, Na Li, Lei Zhang, Fan Zhong, Lihong Chen, Liyan Zeng, Lei Liu

**Affiliations:** 1 Department of Medical and Pharmaceutical Informatics, Hebei Medical University, Shijiazhuang, Hebei, China; 2 School of Basic Medical Science and Intelligent Medicine Institute, Fudan University, Shanghai, China; 3 Department of Rheumatology and Immunology, The Second Hospital of Hebei Medical University, Shijiazhuang, Hebei, China; 4 School of Pharmaceutics, China Medical University-The Queen's University of Belfast Joint College (CQC), Shenyang, Liaoning, China; 5 Department of Clinical Laboratory, The First Affiliated Hospital of Xi'an Jiaotong University, Xi'an, Shaanxi, China; 6 Department of Critical Care Medicine, The First Affiliated Hospital of Xi’an Jiaotong University, Xi'an, Shaanxi, China; 7 International Medical Center, The First Affiliated Hospital of Xi’an Jiaotong University, Xi'an, Shaanxi, China

**Keywords:** biomarker discovery, immune-programmed cell death crosstalk, sepsis-associated encephalopathy, soluble TNFR1, TNFRSF1A

## Abstract

**Background and purpose:**

Sepsis-associated encephalopathy (SAE) is a frequent neurological manifestation of sepsis characterized by acute and diffuse brain dysfunction. Despite increasing evidence of molecular alterations in the septic brain, clinically applicable circulating biomarkers for SAE remain limited. Immune dysregulation and programmed cell death (PCD) pathways have been implicated in SAE pathogenesis, but their translational relevance for peripheral biomarker development remains unclear. This study aimed to identify immune-PCD crosstalk-related biomarkers for SAE through an integrated transcriptomic and clinical evaluation strategy.

**Methods:**

Human brain transcriptomic data from GSE135838 were analyzed using differential expression analysis and gene set enrichment analysis. Immune-related pathways, PCD signatures, and extracellular protein annotations were integrated to prioritize candidate biomarkers with peripheral detection potential. An exploratory cross-species comparison was performed using mouse SAE microglial RNA-sequencing data (GSE198862). Plasma soluble TNFR1 (sTNFR1) was subsequently evaluated in a prospective cohort of 60 septic patients (26 SAE and 34 non-SAE). SAE was defined as acute diffuse cerebral dysfunction during sepsis, identified by reduced GCS and/or a positive CAM-ICU assessment after exclusion of alternative causes, including persistent deep sedation that precluded reliable neurological assessment. Plasma samples for sTNFR1 measurement were collected within 24 h after enrollment, while SAE status was determined during the baseline neurological assessment after ICU admission. Logistic regression, ROC analysis, and bootstrap validation assessed the incremental value of sTNFR1, with 28-day mortality examined exploratorily.

**Results:**

Transcriptomic analysis revealed activation of immune inflammatory responses and multiple PCD-related pathways in SAE brain tissue. Integration of immune, PCD, and differential expression signatures identified 15 immune-PCD crosstalk genes. Extracellular prioritization further identified FAS, IL6, and TNFRSF1A as candidate biomarkers. The immune–PCD signature showed substantial directional concordance in mouse SAE microglial transcriptomes, including concordant increases in Fas and Tnfrsf1a. In the clinical cohort, plasma sTNFR1 levels were significantly elevated in SAE patients compared with non-SAE patients (*p* < 0.001). After adjustment for age and SOFA score, sTNFR1 remained independently associated with SAE. Adding sTNFR1 increased the observed AUC from 0.791 to 0.891; however, the difference did not reach statistical significance by the paired DeLong test (*p* = 0.086). Continuous NRI nevertheless indicated improved reclassification (NRI = 0.950, 95% CI 0.411–1.536; *p* = 0.001). Exploratory analyses also associated SAE and higher sTNFR1 levels with increased 28-day mortality.

**Conclusion:**

This study integrates sepsis-associated brain transcriptomic discovery with prospective peripheral clinical evaluation and identifies circulating sTNFR1 as a candidate biomarker associated with SAE. sTNFR1 may provide additional information beyond conventional severity assessment for SAE identification and merits further validation.

## 1 Introduction

Sepsis is a life-threatening syndrome caused by dysregulated host responses to infection and remains a major global health challenge (1, 2). Recent epidemiological analyses based on the Global Burden of Disease Study estimated that sepsis affected approximately 166 million individuals and was associated with approximately 21.4 million deaths worldwide in 2021, highlighting its persistent disease burden despite advances in critical care management (3). Although improvements in antimicrobial therapy and organ-support strategies have reduced early mortality, neurological complications continue to substantially influence outcomes among critically ill patients with sepsis. Sepsis-associated encephalopathy (SAE) is the most common neurological complication of sepsis and is characterized by acute diffuse cerebral dysfunction occurring in the absence of direct central nervous system infection or other primary neurological disorders (4, 5). The clinical manifestations of SAE are highly heterogeneous, ranging from mild cognitive impairment and delirium to severe consciousness disturbance and coma. Previous studies have demonstrated that SAE is associated with increased mortality, prolonged intensive care unit (ICU) stay, and persistent cognitive impairment after hospital discharge (6). However, SAE diagnosis remains challenging because it relies primarily on neurological assessment and exclusion of alternative causes, including sedative exposure, metabolic disturbances, and pre-existing neurological conditions (4, 7). This challenge is particularly relevant in mechanically ventilated patients who require sedation, in whom reliable neurological assessment may be difficult when sedation cannot be safely reduced or interrupted. In this setting, a blood-based biomarker could provide complementary biological information to support neurological assessment. Currently, no circulating biomarker has been sufficiently validated to provide objective assistance for SAE identification or biological risk stratification. Therefore, identifying clinically accessible biomarkers that reflect SAE-related pathological processes may improve early recognition and facilitate individualized management of patients with sepsis.

The pathogenesis of SAE is increasingly recognized as a complex process involving neuroinflammation, immune dysregulation, blood–brain barrier impairment, and disruption of cellular homeostasis rather than a consequence of systemic inflammation alone (8, 9). The relationship between brain and circulating molecular signals in SAE is likely multifactorial. BBB disruption may facilitate communication between the central and peripheral inflammatory compartments, while systemic and cerebral inflammatory responses may also occur in parallel and generate overlapping molecular signatures in blood and brain. Circulating biomarkers may therefore reflect molecular processes that are shared between systemic and cerebral inflammatory responses in SAE. Programmed cell death (PCD), including apoptosis, necroptosis, pyroptosis, and ferroptosis, is increasingly implicated in the dysregulated host response and organ injury associated with sepsis (10–12). Consistent with this concept, recent studies in sepsis have reported alterations in pathway-associated cell-death markers, including caspase-3 for apoptosis, caspase-1 for pyroptosis, RIPK3 and MLKL for necroptosis, and the ferroptosis-related proteins ACSL4, GPX4, and PTGS2 (13–16). These findings further support the involvement of multiple, potentially interconnected cell-death programs in sepsis (17). However, how immune activation and PCD programs converge in SAE-associated brain alterations, and whether such crosstalk can facilitate the prioritization of clinically accessible biomarkers, remains incompletely understood (18, 19).

To address this gap, we applied an exploratory discovery-to-validation strategy rather than selecting TNFRSF1A *a priori*. We first integrated differential expression, immune- and programmed cell death (PCD)-related gene sets, and extracellular-protein annotations in post-mortem human brain transcriptomes from sepsis and non-sepsis critical-illness controls. The biological consistency of prioritized candidates was then examined in an independent mouse SAE microglial transcriptomic dataset, followed by prospective plasma evaluation in clinically characterized patients with sepsis. This stepwise workflow prioritized TNFRSF1A as a leading extracellular candidate. TNFRSF1A encodes TNFR1, which links TNF-dependent inflammatory signaling with apoptosis and necroptosis and can undergo ectodomain shedding to generate soluble TNFR1 (sTNFR1), providing a biologically plausible circulating readout of TNFR1-pathway activation (20). TNFR1 shedding has been demonstrated in peripheral immune and endothelial cells, while neurons and microglia are also capable of releasing sTNFR1 under inflammatory conditions (21–23). In SAE, disruption of the blood–brain barrier and concurrent neuroinflammatory activation may strengthen the coupling between central and systemic TNFR1-related responses (4, 24), making circulating sTNFR1 a biologically plausible peripheral readout of SAE-associated inflammatory activity. However, the extent to which CNS-derived sTNFR1 directly contributes to plasma levels remains uncertain. We therefore investigated whether plasma sTNFR1 was associated with SAE and whether it provided additional information beyond conventional clinical severity assessment.

## 2 Methods

### 2.1 Transcriptomic data acquisition and differential expression analysis

Human brain transcriptomic data were obtained from the Gene Expression Omnibus (GEO) database (GSE135838). The dataset included 24 post-mortem parietal cortex tissue samples, consisting of 12 sepsis cases and 12 non-septic control subjects who died of noninfectious critical illness during hospitalization. Subjects with acute structural brain injury or indeterminate causes of death were excluded, and the groups were balanced for age, neuropathology, dementia status, and RNA integrity (25). Normalized expression matrices and sample annotations were downloaded from GEO and analyzed using R software. Differential expression analysis between SAE and control samples was performed using the limma package. Genes with |log2 fold change (FC)| > 0.3 and nominal *p* < 0.05 were considered exploratory differentially expressed candidates for downstream biological prioritization. Given the modest sample size and the exploratory aim of candidate discovery, this relatively permissive threshold was used to retain potentially relevant signals for subsequent integrative filtering rather than to define statistically definitive differential expression. Benjamini–Hochberg adjusted *p* values were calculated and reported for transparency but were not used as the primary screening criterion. Candidate prioritization was subsequently constrained by integration with immune- and PCD-related gene sets and extracellular-protein annotations.

### 2.2 Functional enrichment analysis of immune and programmed cell death pathways

Gene set enrichment analysis (GSEA) was performed using a pre-ranked approach based on moderated t-statistics obtained from limma. The Hallmark gene sets from the Molecular Signatures Database (MSigDB) were used as reference pathways. GSEA was performed using the clusterProfiler package, with gene sets containing fewer than 10 or more than 500 genes excluded. Pathway enrichment was evaluated according to normalized enrichment scores (NES) and nominal *p* values. To characterize immune activation and PCD-related alterations in SAE, representative immune pathways were selected from enriched Hallmark pathways, including TNFα signaling via NF-κB, interferon responses, IL6/JAK/STAT3 signaling, complement activation, and inflammatory response. PCD-related gene sets, including apoptosis, necroptosis, ferroptosis, and pyroptosis, were collected from KEGG, FerrDb, and MSigDB Gene Ontology Biological Process datasets. These immune- and PCD-related pathways were further evaluated using pre-ranked GSEA.

### 2.3 Identification of immune-PCD crosstalk genes

To identify SAE-associated genes involved in immune-PCD interactions, an integrative intersection strategy was applied. Exploratory differentially expressed candidates identified by limma were intersected with immune-related and PCD-related gene sets. Immune genes were derived from representative inflammatory pathways described above, while PCD genes included apoptosis, necroptosis, ferroptosis, and pyroptosis-associated genes. Genes overlapping among differentially expressed genes, immune-related genes, and PCD-related genes were defined as immune-PCD crosstalk genes. The intersection process was visualized using Venn diagrams. Expression patterns of these genes were evaluated using Z-score normalized expression values. Heatmaps were generated to compare SAE and control groups, and Pearson correlation analysis was performed to explore co-expression relationships among immune-PCD crosstalk genes.

### 2.4 Extracellular protein prioritization and candidate biomarker identification

Given the need for clinically accessible biomarkers, the immune-PCD crosstalk genes were further prioritized according to their extracellular localization and secretion potential. Extracellular protein annotations were obtained from UniProt, and predicted secreted proteins were obtained from the Human Protein Atlas (HPA). The intersection between immune-PCD crosstalk genes and extracellular/secreted protein-related genes was defined as extracellular candidate genes. Expression differences of these candidate genes between SAE and control samples were assessed using normalized expression values. Candidate genes showing differential expression and extracellular accessibility were prioritized for further evaluation. Among these candidates, TNFRSF1A was selected as the primary candidate biomarker based on its biological relevance in TNF signaling, inflammatory regulation, programmed cell death pathways, and potential detectability in circulation.

### 2.5 Candidate biomarker evaluation and transcriptomic model exploration

The ability of candidate genes to distinguish SAE-related transcriptional patterns was explored using logistic regression models. Expression levels of FAS, IL6, and TNFRSF1A were extracted from the normalized expression matrix. Individual logistic regression models were constructed for each candidate gene, followed by a combined model incorporating all three genes. Because of the limited sample size, model evaluation was performed using leave-one-out cross-validation (LOOCV). In each iteration, one sample was withheld as the testing sample and the remaining samples were used for model training. Cross-validated predicted probabilities were subsequently used for receiver operating characteristic (ROC) analysis. Model performance was evaluated by the area under the ROC curve (AUC) with 95% confidence intervals. For the combined model, the optimal cutoff value was determined using the Youden index, and sensitivity, specificity, and accuracy were calculated. These transcriptomic analyses were considered exploratory and were used for candidate prioritization rather than clinical diagnostic evaluation.

### 2.6 Exploratory cross-species comparison using mouse SAE microglial RNA sequencing

An independent mouse SAE microglial RNA-sequencing dataset (GSE198862) was used for an exploratory cross-species comparison. Microglia were selected as a biologically relevant CNS immune-cell compartment because microglial activation is an important component of SAE-associated neuroinflammatory responses. GSE198862 contains isolated microglia obtained after polymicrobial sepsis induced by peritoneal contamination and infection (PCI); the present analysis included samples collected at day 3 from four PCI-SAE mice and four sham controls. Given the prominent immune-inflammatory signals identified in the human SAE brain transcriptomic analysis, this dataset was used to examine whether candidate genes identified in bulk human cortical tissue showed concordant directional changes within a disease-relevant CNS immune-cell population. This analysis was intended as a supportive and directional cross-species comparison rather than as validation of a cell-type-specific human signature or inference of the cellular origin of the bulk-tissue signal. Differential expression analysis was performed using the edgeR and limma-voom workflow after standard preprocessing. Candidate genes, including Fas, Il6, and Tnfrsf1a, were compared between the human and mouse datasets, and genes showing concordant expression directions were considered directionally consistent across the two transcriptomic contexts. The overall directional concordance of the 15 immune–PCD crosstalk genes was summarized as the proportion of genes showing consistent expression directions between the human and mouse datasets. For pyroptosis-related analysis, mouse Casp4 (historically referred to as Casp11) was analyzed according to current gene annotation.

### 2.7 Prospective clinical cohort and data collection

This prospective observational study consecutively enrolled adult patients with sepsis admitted to the Department of Intensive Care Medicine at the First Affiliated Hospital of Xi’an Jiaotong University between March and May 2026. Sepsis was defined according to the Sepsis-3 criteria. SAE was defined as acute diffuse cerebral dysfunction occurring during sepsis after exclusion of alternative causes, and was identified based on decreased consciousness (GCS reduction) and/or positive CAM-ICU assessment. Patients were excluded if they were aged <18 years, had primary neurological diseases, direct central nervous system infection, severe metabolic encephalopathy, intoxication, persistent deep sedation preventing reliable neurological assessment, or missing key clinical or biomarker data. For SAE classification, neurological assessments were performed as part of routine ICU care. No standardized sedation interruption or prespecified sedation-free assessment window was mandated by the study protocol, and detailed information on the timing and dose of sedative exposure relative to neurological assessment was not systematically available. Therefore, the potential effects of intermittent or moderate sedation could not be fully controlled during SAE classification. A total of 60 patients were included, including 26 patients with SAE and 34 non-SAE patients. Demographic characteristics, disease severity scores, laboratory parameters, organ support therapies, and clinical outcomes were collected. The patient selection process is illustrated in Supplementary Figure S1.

### 2.8 Blood collection and plasma sTNFR1 measurement

Peripheral venous blood samples were collected within 24 h after enrollment. Blood samples were centrifuged at 2,000 × g for 15 min at 4 °C, and plasma aliquots were separated and stored at −80 °C until analysis. Repeated freeze–thaw cycles were avoided. Plasma sTNFR1 concentrations were measured using a human TNFRSF1A ELISA kit (Lilay Biotechnology, China) according to the manufacturer’s instructions. Before analysis, plasma samples were pre-diluted 20-fold with the provided sample diluent. Subsequently, 10 μL of the diluted sample was added to each well together with 40 μL of sample diluent, resulting in a final dilution factor of 100-fold. Absorbance was measured at 450 nm using a Sunrise F50 microplate reader. Plasma sTNFR1 concentrations were calculated from the standard curve and corrected by the total dilution factor, with results expressed as pg/mL. All samples were analyzed in duplicate. Samples with an intra-assay coefficient of variation (CV) > 15% were re-assayed. All measurements included in the analysis were within the valid range of the standard curve.

### 2.9 Clinical phenotype and exploratory outcome definitions

SAE status was considered the primary clinical phenotype and grouping variable in this study. All patients underwent neurological assessment during the baseline evaluation period after ICU admission, with SAE status determined at this stage according to the predefined criteria. For the biomarker analysis, peripheral venous blood samples were collected within 24 h after enrollment. Exploratory clinical outcomes included 28-day all-cause in-hospital mortality and hospitalization status. The time origin for outcome assessment was defined as ICU admission. Twenty-eight-day mortality was defined as in-hospital death occurring within 28 days after ICU admission. Time to death was calculated from ICU admission to the date of death. Patients who died after 28 days were not considered as 28-day mortality events. At day 28, patients were further categorized into three mutually exclusive clinical states: in-hospital death, alive discharge, or ongoing hospitalization, for exploratory outcome analyses.

### 2.10 Statistical analysis

All clinical data were subjected to quality control and completeness assessment before statistical analysis. Continuous variables were summarized as mean ± standard deviation or median (interquartile range, IQR) according to their distribution, while categorical variables were presented as counts and percentages. Distributional characteristics were assessed using the Shapiro–Wilk test and visual inspection of Q-Q plots. Skewed continuous variables used in regression analyses were log-transformed when appropriate. Potential outliers were identified using the IQR method and reviewed against clinical records; clinically plausible values were retained. Baseline characteristics between SAE and non-SAE groups were compared using Welch’s t-test or Mann–Whitney U test for continuous variables and Pearson’s χ^2^ test or Fisher’s exact test for categorical variables.

Binary logistic regression models were constructed to evaluate the association between plasma sTNFR1 levels and SAE status. Given the limited sample size and 26 SAE cases, model complexity was intentionally restricted to a maximum of three prespecified predictors to reduce the risk of overfitting. Variables were selected *a priori* based on clinical relevance rather than univariable statistical significance. Accordingly, the fully adjusted model (Model 2) included age, SOFA score, and log₂-transformed sTNFR1, with no data-driven variable selection. Model performance was interpreted as an internal evaluation within this cohort, and Harrell bootstrap resampling with 2,000 repetitions was used to estimate optimism-corrected performance.

Three models were predefined:

Model 0: SAE ~ log₂(sTNFR1)

Model 1: SAE ~ age10 + SOFA

Model 2: SAE ~ age10 + SOFA + log₂(sTNFR1)

where age10 represents age divided by 10. The effect estimate of log₂(sTNFR1) was interpreted as the odds ratio associated with each doubling of plasma sTNFR1 concentration.

Model performance was evaluated using the area under the receiver operating characteristic curve, Brier score, calibration intercept, and calibration slope. Internal validation was performed using Harrell bootstrap resampling with 2,000 repetitions, and optimism-corrected performance estimates were reported. Incremental improvement of nested models was assessed using likelihood ratio tests. Differences in AUCs were evaluated using the DeLong test, and bootstrap resampling was used to estimate 95% confidence intervals for ΔAUC. Net reclassification improvement (NRI), integrated discrimination improvement (IDI), and decision curve analysis were considered exploratory measures of incremental value. All statistical tests were two-sided, and *p* < 0.05 was considered statistically significant. Statistical analyses were performed using R software.

### 2.11 Sensitivity analyses and exploratory outcomes

Sensitivity analyses were performed to evaluate the robustness of the association between sTNFR1 and SAE. First, Model 2 was additionally adjusted for eGFR to account for potential renal function-related effects. Second, analyses were repeated after excluding patients who received CRRT before blood sampling. Exploratory outcome analyses evaluated the associations of SAE status and sTNFR1 levels with short-term clinical outcomes. Twenty-eight-day mortality was analyzed using competing risk methods, with alive discharge considered as a competing event. Cumulative incidence functions and Fine-Gray models were applied. Because of the limited number of mortality events, Firth penalized logistic regression was used to explore the association between sTNFR1 and 28-day mortality after adjustment for SOFA score. The Firth model converged within the prespecified maximum number of iterations, and profile-likelihood confidence intervals were reported.

### 2.12 Ethical considerations

This prospective observational study was conducted in accordance with the Declaration of Helsinki and was approved by the Ethics Committee of the First Affiliated Hospital of Xi’an Jiaotong University. This study prospectively enrolled patients and used residual plasma samples collected during routine clinical care, without additional blood sampling or intervention solely for research purposes. The requirement for informed consent was waived by the ethics committee because only de-identified residual specimens and clinical data were analyzed.

## 3 Result

### 3.1 Transcriptomic profiling reveals immune-PCD activation in SAE brain tissue

Based on the GSE135838 brain transcriptomic dataset, differential expression analysis was performed between SAE-associated septic brain tissues and non-septic controls. Using an exploratory threshold of |log2FC| > 0.3 and nominal *P* < 0.05, 337 exploratory differentially expressed candidates were identified (Figure 1a). Several genes involved in inflammatory regulation and programmed cell death, including TNFRSF1A, IL6, FAS, CASP4 and CASP7, showed altered expression patterns, suggesting dysregulation of immune and cell death-related processes in SAE. Gene set enrichment analysis based on Hallmark gene sets revealed prominent enrichment of immune-inflammatory pathways, including the IFN-γ response, TNFα/NF-κB signaling, IL6/JAK/STAT3 signaling, IFN-α response, inflammatory response, and complement activation, together with enrichment of the apoptosis pathway (Figure 1b). To determine whether PCD-related alterations extended beyond apoptosis, we further evaluated predefined gene sets representing apoptosis, necroptosis, pyroptosis, and ferroptosis using pre-ranked GSEA. This analysis revealed enrichment signals involving all four PCD programs (Figure 1c). At the gene level, these enrichment signals were driven by leading-edge subsets comprising 69 genes for apoptosis (e.g., TNFRSF1A, FAS, BAX, and CASP1), 71 genes for necroptosis (e.g., MLKL, RIPK1, and GSDMD), 156 genes for pyroptosis (e.g., CASP4, GSDMD, AIM2, and NLRC4), and 35 genes for ferroptosis (e.g., SLC7A11, SAT1, HMOX1, and ALOX5). These findings indicate concurrent enrichment of immune-inflammatory and multiple PCD-related transcriptional programs in sepsis-associated brain tissue.

**Figure 1:**
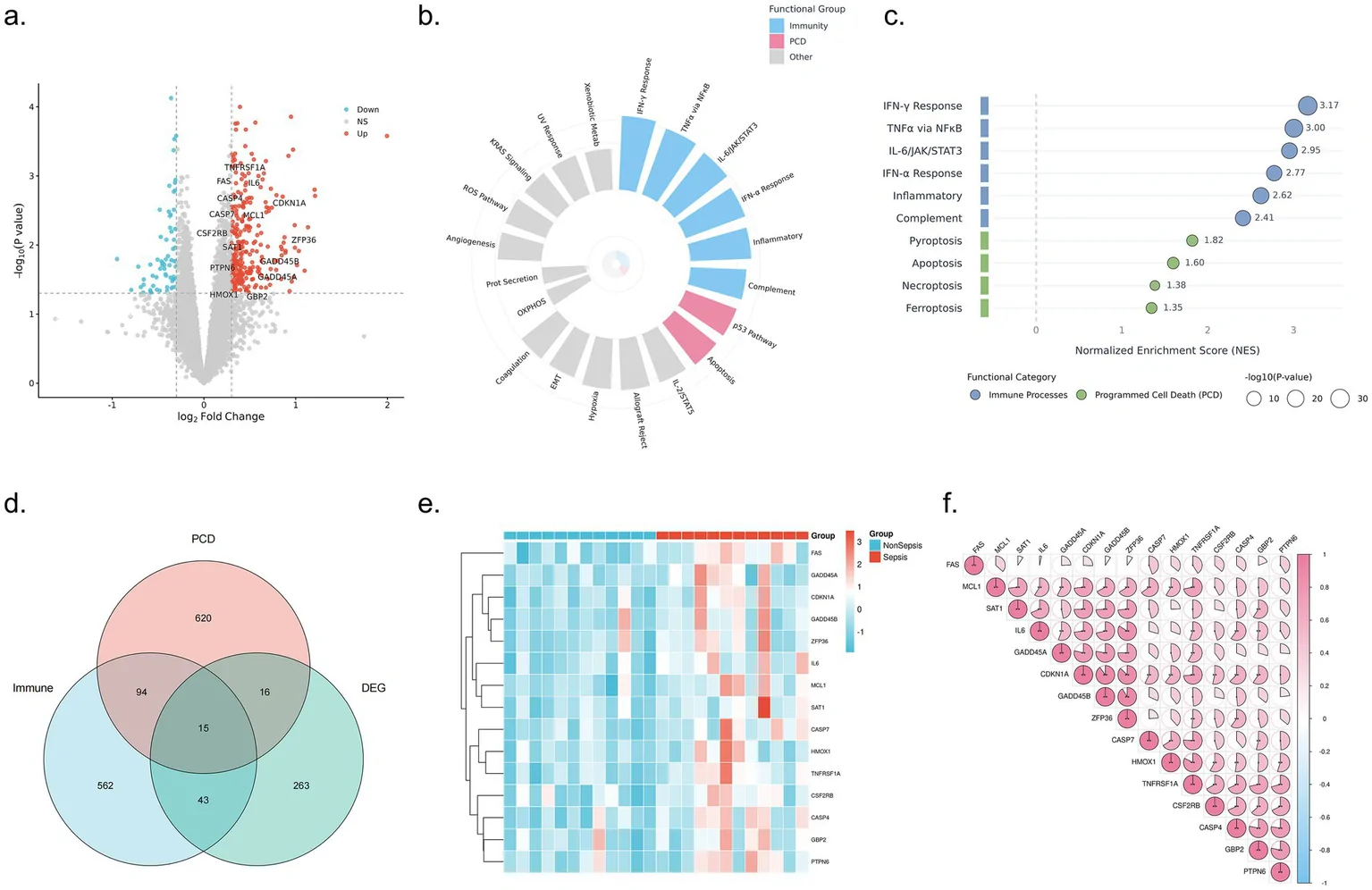
Transcriptomic profiling reveals immune–programmed cell death crosstalk in SAE-associated brain tissue. **(a)** Volcano plot of differentially expressed genes between SAE-associated septic brain tissues and non-septic controls in the GSE135838 dataset. **(b)** Circular plot of representative Hallmark gene set enrichment analysis results, grouped into immune-related, PCD-related, and other biological processes. **(c)** Pre-ranked GSEA of representative Hallmark immune-inflammatory pathways and predefined PCD-related gene sets, including apoptosis, necroptosis, pyroptosis, and ferroptosis, in GSE135838 (SAE-associated septic brain tissues vs. non-septic controls). Bubble size represents −log10(nominal *p* value), and the normalized enrichment score (NES) indicates the direction and magnitude of enrichment. **(d)** Venn diagram showing the intersection of differentially expressed genes (|log2FC| > 0.3, nominal *P* < 0.05), immune-related genes from representative Hallmark immune-inflammatory pathways, and PCD-related genes pooled across predefined apoptosis, necroptosis, pyroptosis, and ferroptosis gene sets, yielding 15 immune–PCD crosstalk genes in GSE135838. **(e)** Heatmap showing the expression patterns of immune–PCD crosstalk genes across SAE-associated and control samples. **(f)** Pearson correlation matrix showing co-expression relationships among the identified immune-PCD crosstalk genes.

### 3.2 Identification and characterization of immune-PCD crosstalk genes in SAE

To identify molecular mediators linking immune activation and programmed cell death, immune-related and PCD-related gene sets were integrated with differentially expressed genes. Immune-related genes were derived from six representative immune Hallmark pathways, while PCD-related genes were obtained from apoptosis, necroptosis, ferroptosis and pyroptosis-associated gene sets. The intersection analysis identified 15 immune-PCD crosstalk genes (Figure 1d), including FAS, IL6, TNFRSF1A, CASP4, CASP7, CDKN1A, GADD45A and others. Expression profiling demonstrated coordinated upregulation of these genes in SAE brain tissues (Figure 1e). Correlation analysis further revealed extensive positive correlations among these genes, suggesting the existence of a coordinated immune-PCD molecular module associated with SAE pathogenesis (Figure 1f).

### 3.3 Extracellular prioritization identifies three candidate circulating biomarkers

To identify clinically accessible biomarkers, the 15 immune–PCD crosstalk genes were further prioritized according to extracellular localization and secretion potential. Integration of UniProt extracellular protein annotations with secretome information from the Human Protein Atlas identified three extracellular candidates: FAS, IL6, and TNFRSF1A (Figure 2a). All three genes showed higher expression in SAE brain tissues than in non-septic controls (Figure 2b). These candidates were subsequently evaluated for transcriptomic discrimination and cross-species directional concordance.

**Figure 2:**
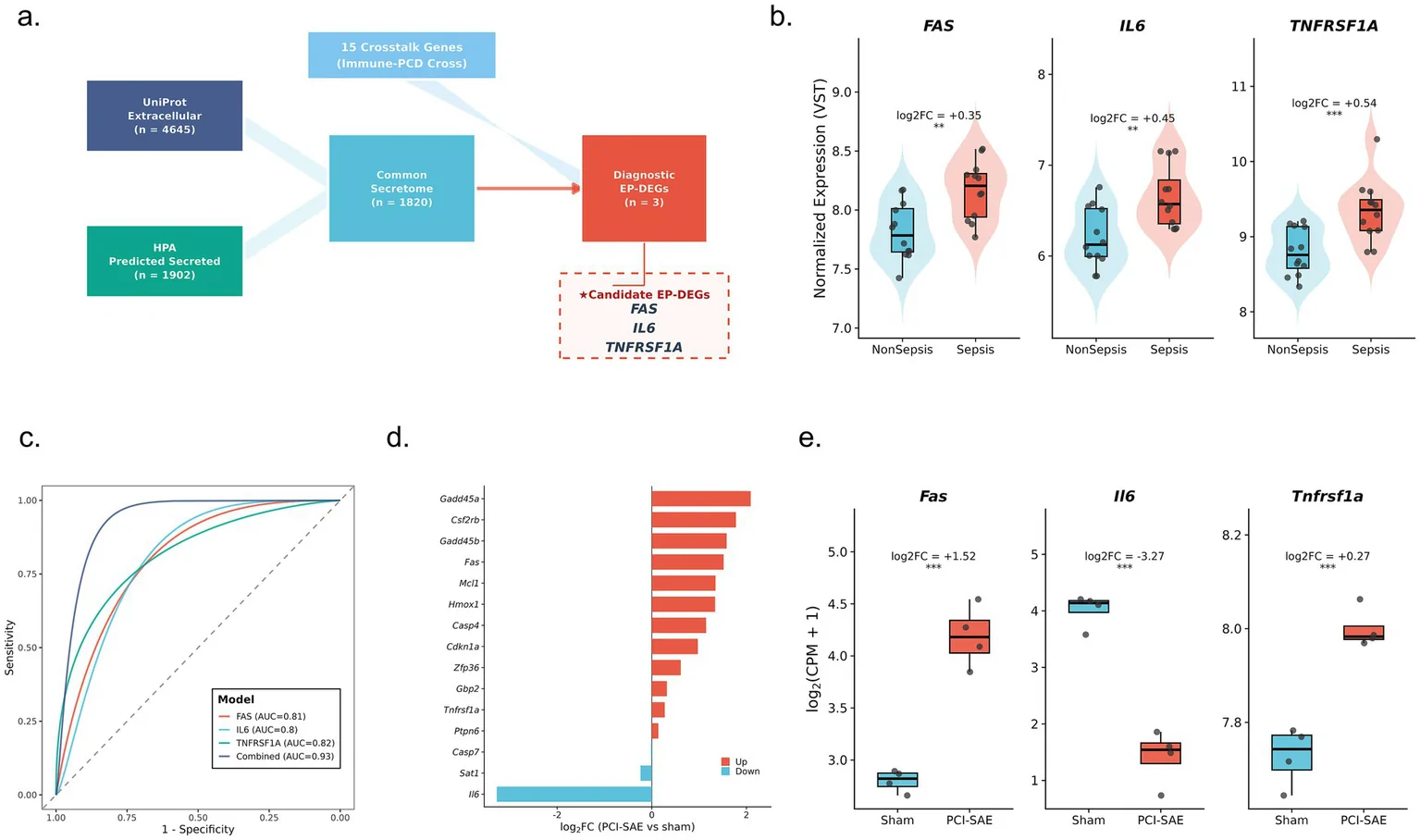
Prioritization and cross-species assessment of extracellular immune–PCD candidate biomarkers. **(a)** Workflow integrating 15 immune-PCD crosstalk genes with extracellular protein annotations from UniProt and secretome-related information from the Human Protein Atlas (HPA), identifying FAS, IL6, and TNFRSF1A as candidate biomarkers. **(b)** Expression of the three candidates in human brain tissues from GSE135838. **(c)** Receiver operating characteristic curves for individual genes and the combined logistic regression model evaluated using leave-one-out cross-validation. **(d)** Exploratory cross-species comparison in mouse SAE microglia from GSE198862. Bars show the log₂ fold changes in the PCI-SAE group relative to the sham group. **(e)** Expression of Fas, Il6, and Tnfrsf1a in mouse microglia from GSE198862. **p < 0.01, and ***p < 0.001. SAE, sepsis-associated encephalopathy; PCD, programmed cell death; ROC, receiver operating characteristic; HPA, Human Protein Atlas.

### 3.4 Transcriptomic discrimination and cross-species comparison prioritize TNFRSF1A

The exploratory discriminative performance of the three extracellular candidates was assessed using the SAE brain transcriptomic dataset. Given the limited sample size, leave-one-out cross-validation (LOOCV) was used for internal evaluation. The individual genes showed moderate discrimination, with AUCs of 0.812 (95% CI, 0.640–0.985) for FAS, 0.778 (95% CI, 0.583–0.972) for IL6, and 0.812 (95% CI, 0.639–0.986) for TNFRSF1A. The combined three-gene model yielded an AUC of 0.903 (95% CI, 0.777–1.000), with 83.3% sensitivity, 91.7% specificity, and 87.5% accuracy at the optimal cutoff (Figure 2c). These findings suggest that the three-gene panel captured SAE-associated transcriptional differences in this exploratory dataset, while the limited sample size precludes firm conclusions regarding its diagnostic performance.

Cross-species directional concordance was further examined using the mouse SAE microglial RNA-sequencing dataset GSE198862. Of the 15 immune–PCD crosstalk genes identified in human brain tissue, 12 (80.0%) showed concordant expression directions in the mouse microglial dataset (Figure 2d), indicating substantial directional overlap between the human bulk-tissue signature and the microglial response to experimental sepsis. Among the three extracellular candidates, Fas and Tnfrsf1a showed concordant increases across the two transcriptomic contexts, whereas Il6 showed an opposite direction in mouse microglia (Figure 2e). These findings provided supportive cross-species evidence for candidate prioritization but were not interpreted as cell-type-specific validation of the human brain transcriptomic signature.

### 3.5 Clinical characteristics of the prospective cohort

A total of 86 consecutive patients with sepsis were screened, of whom 60 with available plasma sTNFR1 measurements and complete clinical data were included in the final analysis (Supplementary Figure S1). The cohort comprised 26 patients with SAE and 34 without SAE. Baseline characteristics are summarized in Table 1.

**Table 1:** Baseline characteristics of patients with and without sepsis-associated encephalopathy (SAE).

Characteristic	Non-SAE (*n* = 34)	SAE (*n* = 26)	*p* value
Demographics			
Age, years	54.2 (14.1)	62.9 (16.1)	**0.034**
Male sex, *n* (%)	24 (70.6)	18 (69.2)	1.000
Body mass index, kg/m^2^	23.1 (21.1–24.6)	24.1 (20.9–27.8)	0.531
Comorbidities			
Hypertension, *n* (%)	19 (55.9)	14 (53.8)	1.000
Diabetes mellitus, *n* (%)	11 (32.4)	11 (42.3)	0.601
Infection site, *n* (%)			
Pulmonary	18 (52.9)	18 (69.2)	0.325
Abdominal	4 (11.8)	0 (0.0)	
Gastrointestinal	1 (2.9)	2 (7.7)	
Bloodstream	9 (26.5)	5 (19.2)	
Urinary	2 (5.9)	1 (3.8)	
Severity scores			
SOFA score	5.8 (2.0)	8.9 (4.2)	**0.004**
APACHE II score	16.0 (14.0–20.0)	23.5 (20.2–26.8)	**<0.001**
NUTRIC score	4.4 (1.8)	5.8 (1.7)	**0.005**
Organ support			
Mechanical ventilation, *n* (%)	7 (20.6)	22 (84.6)	**<0.001**
Vasopressor use, *n* (%)	4 (11.8)	13 (50.0)	**0.002**
CRRT, *n* (%)	4 (11.8)	4 (15.4)	0.717
Inflammatory and laboratory markers			
White blood cell count, ×10^9^/L	9.6 (5.6)	10.7 (6.3)	0.442
Neutrophil-to-lymphocyte ratio	8.52 (3.90–18.76)	7.58 (3.91–17.18)	0.660
Platelet count, ×10^9^/L	165.5 (89.0–257.0)	131.5 (89.5–212.5)	0.314
Hemoglobin, g/L	95.0 (81.2–104.0)	82.0 (78.2–91.2)	**0.032**
Lactate, mmol/L	1.1 (0.9–1.4)	1.2 (0.9–1.9)	0.246
PaO₂/FiO₂, mmHg	275.4 (96.8)	319.7 (104.1)	0.099
Procalcitonin, ng/mL	0.73 (0.21–2.21)	0.80 (0.18–2.09)	0.765
Interleukin-6, pg/mL	23.80 (7.64–53.95)	37.00 (29.45–110.88)	**0.030**
Albumin, g/L	31.1 (4.2)	30.1 (4.2)	0.369
Aspartate aminotransferase, U/L	25.0 (18.2–35.0)	27.5 (17.5–79.2)	0.374
D-dimer, mg/L	5.6 (6.5)	6.8 (9.0)	0.615
Renal function			
Estimated glomerular filtration rate, mL/min/1.73 m^2^	86.1 (32.0–116.0)	65.2 (37.6–95.8)	0.293
Circulating biomarker			
Plasma sTNFR1, pg/mL	4090.3 (3299.5–4921.0)	5855.5 (5026.9–6790.3)	**<0.001**
Outcomes			
Hospital length of stay, days	22.5 (13.0–34.8)	18.5 (11.5–30.0)	0.328
28-day mortality, *n* (%)	3 (8.8)	10 (38.5)	**0.010**

SAE, sepsis-associated encephalopathy; SOFA, Sequential Organ Failure Assessment; APACHE II, Acute Physiology and Chronic Health Evaluation II; NUTRIC, Nutrition Risk in the Critically Ill; CRRT, continuous renal replacement therapy; PaO₂/FiO₂, ratio of arterial oxygen partial pressure to fractional inspired oxygen; sTNFR1, soluble tumor necrosis factor receptor 1. Bold values indicate statistically significant differences (*p* < 0.05).

Compared with the non-SAE group, patients with SAE were older (62.9 ± 16.1 vs. 54.2 ± 14.1 years, *p* = 0.034) and had higher SOFA (8.9 ± 4.2 vs. 5.8 ± 2.0, *p* = 0.004), APACHE II (23.5 [20.2–26.8] vs. 16.0 [14.0–20.0], *p* < 0.001), and NUTRIC scores (5.8 ± 1.7 vs. 4.4 ± 1.8, *p* = 0.005). Notably, mechanical ventilation was substantially more frequent in the SAE group (84.6% vs. 20.6%, *p* < 0.001), and vasopressor use was also more common (50.0% vs. 11.8%, *p* = 0.002). Together with the higher severity scores, the greater requirement for organ support was consistent with more severe acute illness in the SAE group and supported prespecified adjustment for SOFA score in subsequent multivariable analyses. Patients with SAE had lower hemoglobin levels (82.0 [78.2–91.2] vs. 95.0 [81.2–104.0] g/L, *p* = 0.032) and higher circulating IL-6 concentrations (37.0 [29.5–110.9] vs. 23.8 [7.6–54.0] pg/mL, *p* = 0.030). No significant between-group differences were observed in white blood cell count, neutrophil-to-lymphocyte ratio, platelet count, lactate, procalcitonin, or renal function indices.

### 3.6 Plasma sTNFR1 levels are elevated in patients with SAE

Plasma sTNFR1 concentrations were significantly higher in patients with SAE than in those without SAE (Figure 3a). The median concentration was 5855.5 pg/mL (IQR, 5026.9–6790.3) in the SAE group and 4090.3 pg/mL (IQR, 3299.5–4921.0) in the non-SAE group (Mann–Whitney U test, *p* < 0.001). In stratified analyses by eGFR, mechanical ventilation, age, and vasopressor use, higher sTNFR1 concentrations were observed in the SAE group across all examined strata (Supplementary Figure S2).

**Figure 3:**
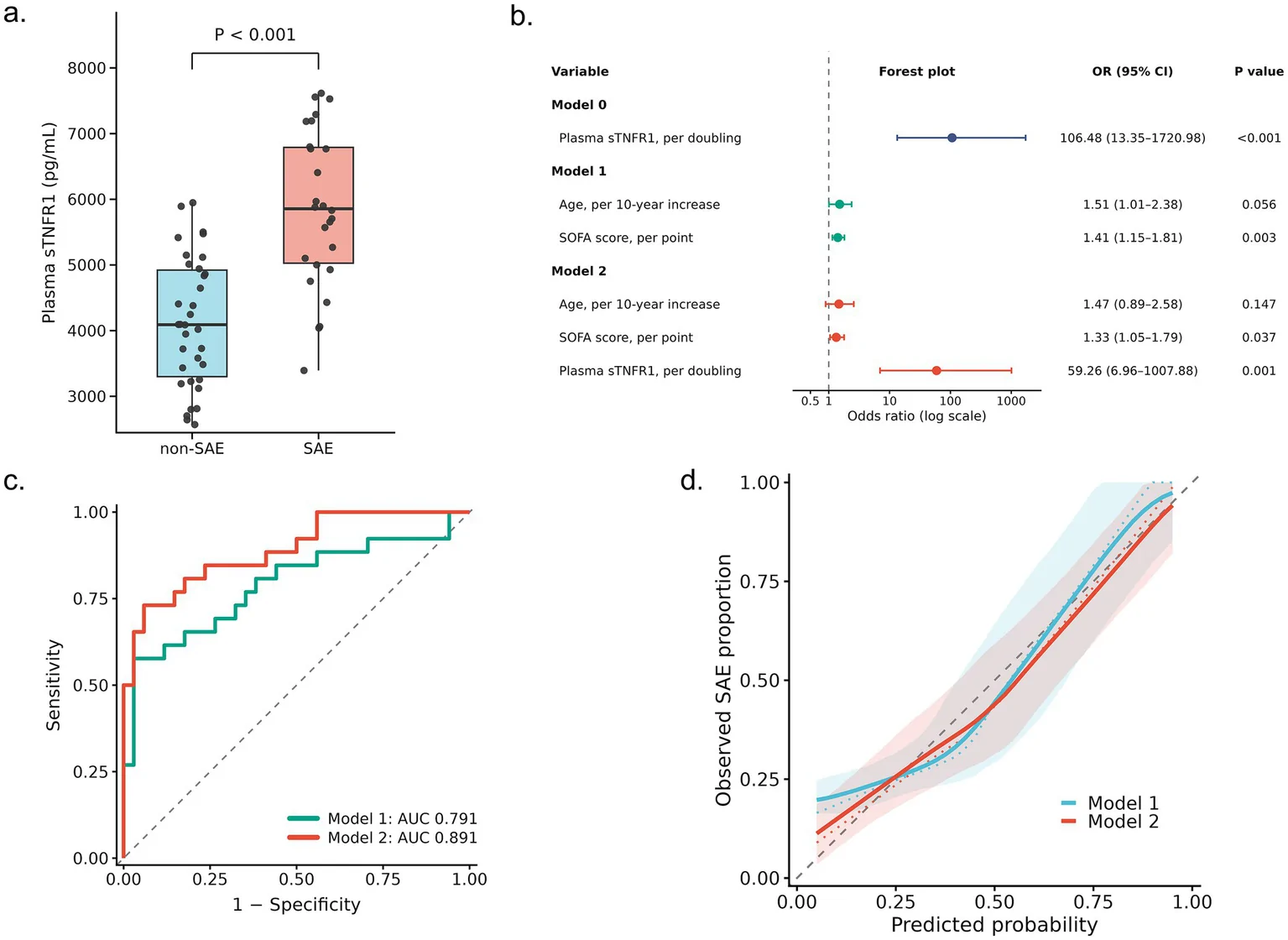
Clinical evaluation of plasma sTNFR1 for SAE identification. **(a)** Plasma sTNFR1 levels in patients with and without SAE. **(b)** Odds ratios for SAE across logistic regression models. Model 0 included log₂-transformed sTNFR1; Model 1 included age per 10-year increase and SOFA score; Model 2 added sTNFR1 to Model 1. The sTNFR1 estimate represents each doubling in concentration. **(c)** ROC curves comparing Model 1 with Model 2. **(d)** Calibration curves comparing predicted and observed SAE probabilities. SAE, sepsis-associated encephalopathy; sTNFR1, soluble tumor necrosis factor receptor 1; SOFA, Sequential Organ Failure Assessment; OR, odds ratio; ROC, receiver operating characteristic.

### 3.7 Incremental value of sTNFR1 beyond age and SOFA score

Three prespecified logistic regression models were constructed to assess whether plasma sTNFR1 provided additional information beyond age and SOFA score (Figure 3b). In the univariable model (Model 0), each doubling of plasma sTNFR1 was associated with higher odds of SAE (OR, 106.48; 95% CI, 13.35–1720.98; *p* < 0.001). In the clinical model comprising age and SOFA score (Model 1), a higher SOFA score was associated with SAE (OR per point, 1.41; 95% CI, 1.15–1.81; *p* = 0.003). After sTNFR1 was added to the clinical model (Model 2), it remained associated with SAE after adjustment for age and SOFA score (OR per doubling, 59.26; 95% CI, 6.96–1007.88; *p* = 0.001). In a sensitivity analysis using Firth penalized logistic regression with the same covariates, the association remained directionally consistent, although the point estimate was attenuated (OR per doubling, 34.60; 95% CI, 5.01–438.29). Given the limited number of SAE events and the wide confidence intervals around the effect estimates, these multivariable results should be interpreted cautiously and regarded as internally evaluated associations rather than externally validated estimates.

The AUC increased from 0.791 for Model 1 to 0.891 for Model 2, corresponding to an observed ΔAUC of 0.101 (Figure 3c). The bootstrap 95% CI for ΔAUC excluded zero (0.012–0.232), whereas the paired DeLong test did not reach statistical significance (*p* = 0.086). Addition of sTNFR1 improved model fit according to the likelihood ratio test (χ^2^ = 17.29, *p* < 0.001). Calibration plots showed reasonable agreement between predicted and observed SAE probabilities for both models (Figure 3d). Reclassification analyses further supported the incremental contribution of sTNFR1, with a continuous NRI of 0.950 (95% CI, 0.411–1.536; *p* = 0.001) and an IDI of 0.207 (95% CI, 0.051–0.437; *p* = 0.046) (Table 2).

**Table 2:** Incremental performance of adding plasma sTNFR1 to the clinical model for identifying sepsis-associated encephalopathy.

Metric	Estimate	95% CI	*p* value
ΔAUC (Model 2 − Model 1)	0.101	0.012 to 0.232	0.086
Continuous NRI	0.950	0.411 to 1.536	**0.001**
Event NRI	0.538	0.200 to 0.833	**0.001**
Non-event NRI	0.412	0.120 to 0.795	**0.018**
IDI	0.207	0.051 to 0.437	**0.046**

AUC, area under the receiver operating characteristic curve; CI, confidence interval; IDI, integrated discrimination improvement; NRI, net reclassification improvement; SAE, sepsis-associated encephalopathy; Bold values indicate statistically significant differences (*p* < 0.05).

### 3.8 Sensitivity analyses and exploratory associations with 28-day outcomes

Sensitivity analyses were performed to examine the robustness of the association between plasma sTNFR1 and SAE (Figure 4a). After additional adjustment for eGFR, sTNFR1 remained associated with SAE (OR per doubling, 80.02; 95% CI, 8.21–1845.56; *p* = 0.001). A consistent association was observed after excluding patients who received CRRT before blood sampling (OR per doubling, 116.98; 95% CI, 8.75–4642.84; *p* = 0.002). Thus, the association was not materially altered by adjustment for renal function or exclusion of patients receiving CRRT.

**Figure 4:**
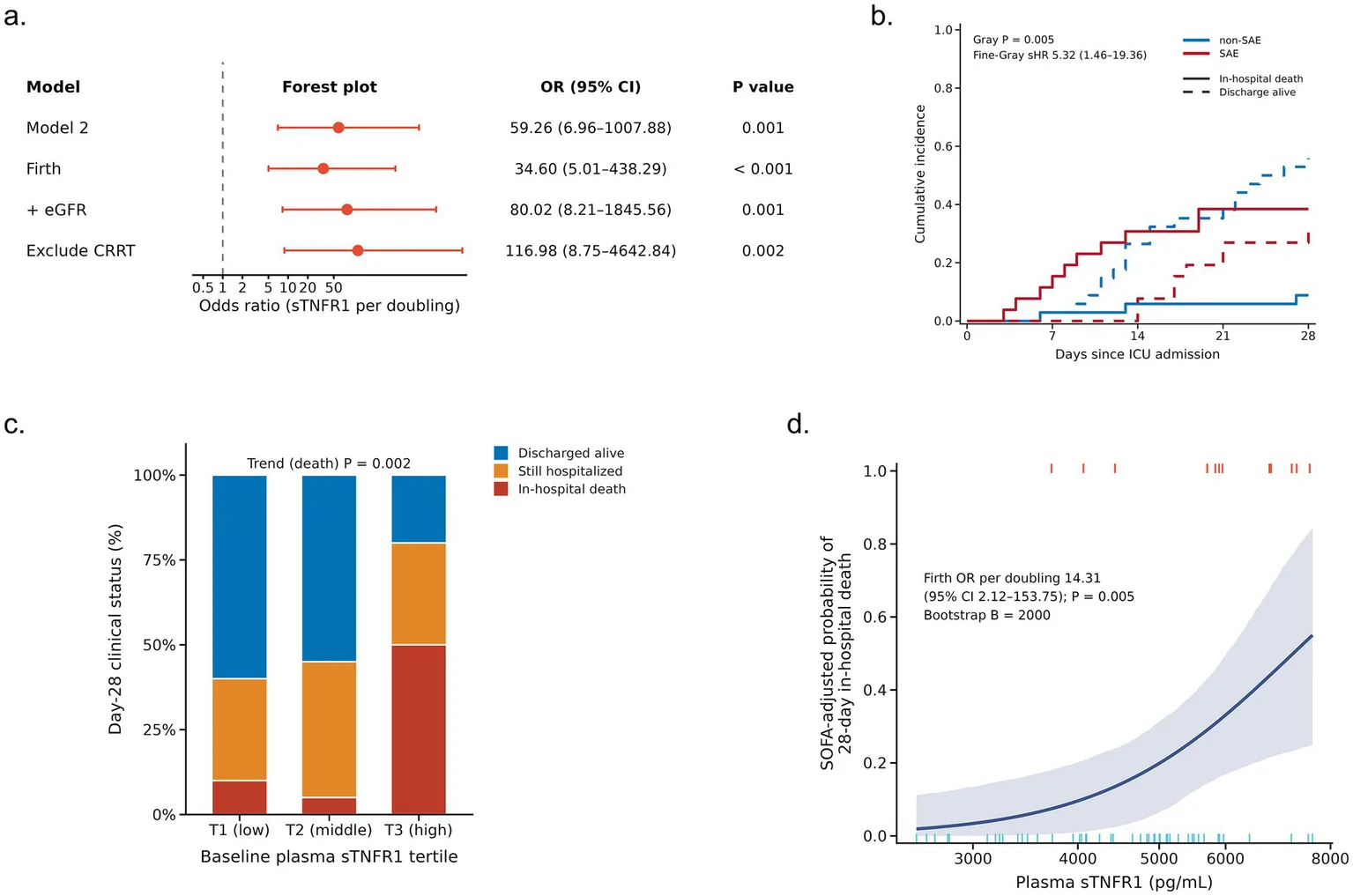
Sensitivity and exploratory outcome analyses of plasma sTNFR1. **(a)** Sensitivity analyses adjusting for eGFR or excluding patients receiving CRRT. Odds ratios represent each doubling of plasma sTNFR1. **(b)** Cumulative incidence of 28-day in-hospital mortality by SAE status, with discharge alive treated as a competing event. **(c)** Twenty-eight-day clinical status across plasma sTNFR1 tertiles. **(d)** SOFA-adjusted probability of 28-day in-hospital mortality estimated using Firth logistic regression. Shading indicates bootstrap 95% confidence intervals. SAE, sepsis-associated encephalopathy; sTNFR1, soluble tumor necrosis factor receptor 1; eGFR, estimated glomerular filtration rate; CRRT, continuous renal replacement therapy; SOFA, Sequential Organ Failure Assessment.

Exploratory analyses examined the associations of SAE and plasma sTNFR1 with 28-day clinical outcomes (Figures 4b–d). Thirteen patients died in hospital within 28 days. The cumulative incidence of mortality was higher in patients with SAE than in those without SAE (38.5% vs. 8.8%; Gray’s test, *p* = 0.005). In Fine–Gray regression, SAE was associated with a higher incidence of 28-day in-hospital mortality (subdistribution HR, 5.32; 95% CI, 1.46–19.36; *p* = 0.011), with discharge alive treated as a competing event. The distribution of 28-day clinical status differed across baseline sTNFR1 tertiles, with the proportion of deaths increasing at higher sTNFR1 levels (trend *p* = 0.002; Figure 4c). In SOFA-adjusted Firth logistic regression based on 13 deaths, each doubling of sTNFR1 was associated with higher odds of 28-day in-hospital mortality (OR, 14.31; 95% CI, 2.12–153.75; *p* = 0.005; Figure 4d). Given the limited number of deaths, particularly after stratification by sTNFR1 tertiles, and the wide confidence interval of the Firth regression estimate, these findings should be interpreted as hypothesis-generating rather than as definitive evidence of prognostic value.

## 4 Discussion

Sepsis-associated encephalopathy is increasingly recognized as a complex neurological syndrome involving neuroinflammation, immune dysregulation, and metabolic disturbances; however, how these molecular alterations within the septic brain can be captured through clinically accessible biomarkers remains unclear (4, 26). In the present study, we identified immune-inflammatory and PCD-related transcriptional alterations in sepsis-associated brain tissue and used these molecular features to prioritize candidates for peripheral evaluation. By integrating human brain transcriptomic profiling with extracellular protein prioritization and clinical evaluation, we identified TNFRSF1A/sTNFR1 as a candidate biomarker for SAE. Our findings extend previous observations of inflammatory activation in SAE by showing overlapping immune-inflammatory and cell-death-related transcriptional signals within the same sepsis-associated brain context (12). Importantly, the elevation of plasma sTNFR1 in patients with SAE and the observed favorable trend in discrimination beyond clinical severity assessment support its further evaluation as a candidate circulating biomarker.

The identification of an immune-PCD crosstalk signature provides a broader perspective on the molecular mechanisms underlying SAE. Previous studies have largely emphasized excessive inflammation, microglial activation, and blood–brain barrier disruption as key contributors to septic brain dysfunction; however, increasing evidence suggests that inflammatory signaling and cell fate regulation are closely interconnected processes rather than independent pathological events (9, 27, 28). In this context, pathways involved in TNF signaling, interferon responses, IL-6/JAK/STAT3 activation, and inflammatory cell death may collectively contribute to sustained neuroimmune activation and neuronal dysfunction during sepsis (29). Our transcriptomic analysis identified an overlapping immune–PCD transcriptional signature in sepsis-associated brain tissue. Many of the immune and cell-death-related genes identified in this analysis have previously been implicated in sepsis or SAE; therefore, the significance of our findings does not lie in the discovery of entirely novel individual markers. Rather, our results show that established inflammatory and PCD-related signals converge within the same sepsis-associated brain transcriptomic context and can be integrated to prioritize candidate molecules with potential translational relevance. Importantly, this transcriptomic overlap should be interpreted as evidence of molecular convergence and co-expression rather than proof of direct regulatory interactions between immune and PCD pathways. Among these immune-PCD crosstalk genes, TNFRSF1A attracted particular attention because of its dual relevance to inflammatory signaling and regulated cell death pathways (30). Its extracellular accessibility further provided a rationale for subsequent peripheral biomarker evaluation.

TNFRSF1A was particularly attractive for translational evaluation because it connects inflammatory signaling with cell fate regulation and produces a soluble extracellular form detectable in peripheral blood (31). TNFR1 is a membrane receptor, and shedding of its extracellular domain generates soluble TNFR1 (sTNFR1), which can be measured as an indicator of TNF receptor system activation (20). Previous studies have associated elevated circulating sTNFR1 levels with sepsis, systemic inflammation, organ dysfunction, and adverse outcomes in critically ill patients (32, 33). The relevance of circulating sTNFR1 may extend beyond acute critical illness, as it has also been investigated in the context of chronic infectious immune dysregulation. In long-term HIV-1 elite controllers, higher plasma sTNFR1 levels were associated with subsequent loss of natural virological control, supporting a broader role of TNF/TNFR1-related signaling across different forms of infectious immune dysregulation (34). Importantly, circulating sTNFR1 has also been linked to acute brain dysfunction during critical illness. Girard et al. reported that higher plasma sTNFR1 concentrations were independently associated with subsequent delirium in mechanically ventilated ICU patients after adjustment for age, illness severity, and sepsis (35), while Ritter et al. similarly found elevated sTNFR1 levels to be independently associated with delirium after adjustment for sepsis and sedation (36). These observations support the biological and clinical plausibility of sTNFR1 as a marker associated with acute brain dysfunction during critical illness. However, delirium in heterogeneous ICU populations is not equivalent to SAE. The significance of the present study therefore lies not in the first identification of sTNFR1 as an inflammation-associated marker of brain dysfunction, but in its independent prioritization through an immune–PCD-oriented brain transcriptomic workflow and subsequent evaluation specifically within a sepsis cohort comparing patients with and without SAE.

An important consideration, however, is that the anatomical source of circulating sTNFR1 cannot be determined from the present study. TNFR1 shedding has been demonstrated in peripheral immune and endothelial cells (21, 37), while neurons and microglia are also capable of releasing sTNFR1 under inflammatory conditions (23). In SAE, blood–brain barrier (BBB) disruption and concurrent neuroinflammatory activation may strengthen the coupling between central and systemic TNFR1-related responses (9). However, direct evidence that endogenous sTNFR1 crosses the disrupted BBB during sepsis is currently lacking. Experimental studies under intact BBB conditions have shown limited brain penetration of soluble TNF receptor constructs (38), although these studies did not specifically examine endogenous sTNFR1 or the septic state. Accordingly, plasma sTNFR1 should not be interpreted as a brain-specific release product, but rather as a biologically plausible peripheral readout of TNFR1-related inflammatory activity associated with SAE, potentially integrating signals from both central and systemic compartments. The extent to which CNS-derived sTNFR1 directly contributes to plasma levels remains uncertain, and paired plasma–CSF measurements together with assessment of BBB integrity will be required to clarify the relative contributions of these sources.

Although the primary objective of this study was to integrate brain transcriptomic discovery with peripheral biomarker evaluation, cross-species analysis provided additional support for the biological relevance of the identified molecular signature. The human brain transcriptomic dataset reflects clinically relevant disease-associated alterations but is limited by tissue heterogeneity and the availability of suitable validation cohorts. Therefore, we further examined the expression patterns of immune-PCD crosstalk genes in an independent mouse SAE microglial dataset. Microglia were selected because they represent a major CNS-resident immune-cell population involved in SAE-associated neuroinflammatory responses, thereby providing a biologically relevant context in which to examine whether candidate-gene changes were reproduced within a disease-relevant immune compartment. The substantial directional concordance observed between the human bulk transcriptomic signature and the mouse microglial response suggests that at least part of the immune–PCD program identified in human SAE is also represented in experimental sepsis-associated microglial activation. Notably, Fas and Tnfrsf1a showed concordant increases across the two transcriptomic contexts, providing additional support for their involvement in SAE-related inflammatory and cell-death signaling, whereas Il6 showed a discordant pattern that may reflect differences in species, cellular composition, disease models, or sampling time points. Importantly, this cross-species comparison should be interpreted as supportive rather than as direct cell-type-specific validation, because the human discovery dataset represents bulk cortical tissue containing mixed cell populations, whereas GSE198862 profiles isolated mouse microglia. Accordingly, the concordant increase in Tnfrsf1a in mouse microglia supports microglial participation in the TNFR1-related response to experimental sepsis but does not imply that microglia are the predominant cellular source of TNFRSF1A in the human septic brain. Despite these biological differences, the reproducible directional changes across distinct experimental contexts strengthen the biological plausibility of the identified immune–PCD signature and further support the prioritization of TNFRSF1A for peripheral biomarker evaluation.

From a clinical perspective, our findings suggest that circulating sTNFR1 may provide molecular information complementary to conventional severity assessment for SAE identification. Current clinical evaluation of SAE remains challenging because it largely relies on neurological examination and clinical severity assessments, which may be affected by sedation status, metabolic disturbances, and the complex clinical environment of critical illness. Therefore, a circulating biomarker providing molecular information complementary to clinical assessment may support the identification and biological characterization of patients with SAE. More broadly, the integrative transcriptomic-to-clinical strategy applied in this study provides a potential framework for using tissue-derived molecular signatures to prioritize clinically accessible circulating biomarkers.

A key strength of this study is its stepwise design, which integrates human brain transcriptomic discovery, extracellular-protein prioritization, independent cross-species biological corroboration, and prospective plasma evaluation, thereby linking molecular discovery with clinical translation across complementary datasets. In addition, the clinical analysis used prespecified multivariable models, bootstrap internal validation, and sensitivity analyses to evaluate the robustness of the association between plasma sTNFR1 and SAE. Several limitations should be considered when interpreting our findings. First, the prospective clinical cohort was relatively small and derived from a single center. Given the 26 SAE events, the multivariable model was intentionally restricted to three prespecified predictors to reduce model complexity. Although bootstrap resampling was used for internal validation, the limited sample size and event count may still affect the stability and precision of the estimated associations and model performance. Accordingly, these clinical findings should be regarded as preliminary and require confirmation in larger, independent multicenter cohorts. The 28-day outcome analyses were based on only 13 deaths. Although Firth penalized logistic regression was used to reduce small-sample bias, the wide confidence interval around the estimated association indicates substantial imprecision. These mortality findings should therefore be interpreted as exploratory and hypothesis-generating. Second, the discovery analysis was based on a publicly available human brain transcriptomic dataset with a limited sample size (*n* = 24), and the exploratory use of a nominal *p*-value threshold with a modest fold-change cutoff may have increased sensitivity at the expense of specificity during initial candidate screening. These factors may influence the stability of candidate selection and pathway-level interpretation. In addition, the supportive cross-species analysis compared bulk human cortical tissue with isolated mouse microglia obtained from an experimental PCI model. Differences in species, tissue context, cellular composition, and sampling conditions limit direct comparability between these datasets. Accordingly, the observed directional concordance should be interpreted as supportive and does not establish cell-type-specific validation or the cellular origin of TNFRSF1A in the human septic brain. Third, SAE classification may have been influenced by sedative exposure and other competing causes of impaired consciousness. Although patients with persistent deep sedation that precluded reliable neurological assessment were excluded, standardized sedation interruptions, prespecified sedation-free assessment windows, and detailed information on sedative dose and timing relative to neurological assessment were not systematically available. This limitation is particularly relevant given the substantially higher frequency of mechanical ventilation in the SAE group, which may have been accompanied by greater exposure to sedative medications. Accordingly, residual misclassification related to intermittent or moderate sedation cannot be excluded, and some neurological abnormalities may have overlapped with drug-induced delirium or coma. Other competing causes of cerebral dysfunction, including hypoxic brain injury, also cannot be completely excluded in this observational cohort. Fourth, paired plasma and cerebrospinal fluid sTNFR1 measurements were not available, and BBB integrity was not directly assessed. Therefore, the relative contributions of central versus systemic or endothelial sources to circulating sTNFR1 cannot be determined from the present study. Finally, the observational design of this study does not establish a causal relationship between TNFRSF1A/sTNFR1 activation and SAE development. Further mechanistic studies are required to determine whether TNFR1-related signaling contributes directly to SAE pathogenesis or primarily reflects downstream immune activation.

Future investigations integrating longitudinal plasma sTNFR1 measurements, larger prospective cohorts, and mechanistic experimental models will be important to clarify its value for SAE prediction, monitoring, and potential biological stratification.

## 5 Conclusion

This study identified coordinated enrichment of immune-inflammatory and programmed cell death-related transcriptional programs in SAE-associated brain tissue and defined a set of immune-PCD crosstalk genes. Through extracellular prioritization and cross-species analysis, TNFRSF1A was identified as a candidate molecule with potential for peripheral validation. In the clinical cohort, plasma sTNFR1 levels were significantly elevated in patients with SAE and remained independently associated with SAE after adjustment for age and SOFA score. The addition of sTNFR1 showed a favorable trend in model discrimination beyond clinical variables. These findings support further evaluation of sTNFR1 as a candidate circulating biomarker associated with SAE, although its cellular and tissue origins and its relationship to central TNFR1 signaling remain to be established. Further validation in larger multicenter cohorts and mechanistic studies is required before its clinical implementation.

## Data Availability

The datasets presented in this study can be found in online repositories. The names of the repository/repositories and accession number(s) can be found: https://www.ncbi.nlm.nih.gov/geo/ (GSE135838, GSE198862).
