# Supplementary material for: Immune-programmed cell death crosstalk implicates plasma sTNFR1 as a candidate biomarker for sepsis-associated encephalopathy
Source: Front Neurol. 2026 Oct 14;17:1954784. doi: 10.3389/fneur.2026.1954784 (PMC13713390; doi:10.3389/fneur.2026.1954784)
Supplement: Supplementary file 1 [file Supplementary_file_1.DOCX]

Supplementary Figure S1. Flow diagram of the clinical validation cohort.

Supplementary Figure S2. Stratified comparisons of plasma sTNFR1 levels in patients with and without SAE. (a) Stratified by eGFR (≥60 or <60 mL/min/1.73 m²). (b) Stratified by mechanical ventilation status. (c) Stratified by age (<65 or ≥65 years). (d) Stratified by vasopressor use. Box plots show plasma sTNFR1 levels with individual data points. Exact P values are indicated. SAE, sepsis-associated encephalopathy; sTNFR1, soluble tumor necrosis factor receptor 1; eGFR, estimated glomerular filtration rate.
